# 紫檀芪抗肿瘤作用机制研究进展

**DOI:** 10.3779/j.issn.1009-3419.2018.12.01

**Published:** 2018-12-20

**Authors:** 晓雁 张, 娇 张, 丽群 徐, 志强 马, 守印 狄, 原 高, 小飞 李, 小龙 闫, 红梅 张

**Affiliations:** 1 710032 西安，第四军医大学西京医院肿瘤科 Department of Clinical Oncology, Xijing Hospital, the Fourth Military Medical University, Xi'an 710032, China; 2 710038 西安，第四军医大学唐都医院胸外科 Department of Thoracic Surgery, Tangdu Hospital, the Fourth Military Medical University, Xi'an 710038, China; 3 710032 西安，第四军医大学学员一旅五营 Battalion 5, the First Brigade of Cadets, the Fourth Military Medical University, Xi'an 710032, China

**Keywords:** 紫檀芪, 肿瘤, 凋亡, 增殖, 侵袭, Peterostilbene, Neoplasms, Apoptosis, Proliferation, Invasion

## Abstract

紫檀芪（3, 5-二甲氧基-4’-羟基二苯乙烯）是一种主要存在于蓝莓、葡萄和花榈木中的多酚类化合物。已有的研究发现紫檀芪具有抗肺癌、乳腺癌、胃癌、结肠癌等多种肿瘤的抗癌作用。其作用机制涉及调控影响多种肿瘤生物学特性。此外，紫檀芪具有比白藜芦醇更高的生物利用度和生物活性，其抗肿瘤作用和机制值得深入探讨和研究。

## Abstract (en)

Pterostilbene (3, 5-dimethoxy-4'-hydroxystilbene) is a polyphenolic compound primarily found in blueberries, grapes, and a tree wood, pterocarpus marsupium. Studies demonstrate that pterostilbene inhibits a variety of cancers, such as lung, breast, stomach, colon, *etc*. The anti-cancer activities are related to the regulation of several hallmarks of cancer. Moreover, pterostilbene exhibits much greater bioavailability and bioactivity than resveratrol which warrants further investigation in the anti-cancer functions and mechanisms.

紫檀芪（3, 5-二甲氧基-4'-羟基二苯乙烯）主要存在于蓝莓、葡萄、花榈木等植物中^[1, 2]^。紫檀芪是白藜芦醇（3, 4', 5-三羟基二苯乙烯）的二甲基衍生物^[3]^，二者具有相似的生物活性，包括抗肿瘤、抗血脂障碍、治疗糖尿病以及心血管和神经保护作用，其机制与抗炎和抗氧化作用相关^[1, 2]^，但紫檀芪较白藜芦醇的抗氧化和抗肿瘤活性作用更强^[1]^。由于第3和第5位的羟基替换为甲氧基，紫檀芪与白藜芦醇相比，其亲脂性和口服吸收率增加，半衰期延长，因而具有更高的生物利用度^[4]^。研究发现使用较低浓度剂量的紫檀芪（5 μmol/L-10 μmol/L）后，具有清除活性氧簇的功效而发挥抗紫外线诱导皮肤癌发生的作用^[5]^。此外，当紫檀芪维持在相对高浓度剂量（> 20 μmol/L）时则具有显著的抗肿瘤作用^[6-9]^，包括诱导肿瘤细胞凋亡^[2, 7, 10-13]^，抑制肿瘤细胞增殖^[1, 2, 11, 14-16]^、侵袭和转移^[17-20]^及血管生成^[13, 17, 21]^，抑制肿瘤干细胞特性^[15, 22-25]^。

## 1 紫檀芪与肿瘤细胞增殖

肿瘤细胞最基本的特征就是失去调控而可以无限增殖^[26]^，而紫檀芪可作用于多种信号通路进而抑制肿瘤细胞增殖^[2, 14-16]^。研究表明，高浓度紫檀芪（50 μmol/L）诱导G_1_期阻滞，低浓度紫檀芪（< 10 μmol/L）诱导S期阻滞，但其机制仍不清楚^[16]^。紫檀芪上调细胞周期蛋白依赖性激酶（cyclin-dependent kinase, CDK）抑制物p53、p21、p27和p16蛋白表达，从而抑制CDK2/4/6和细胞周期蛋白cyclin A、cyclin E表达，导致胃癌AGS细胞的细胞周期阻滞于G_0_期/G_1_期，但cyclin D1表达未受影响^[2, 27]^。低浓度紫檀芪激活毛细血管扩张共济失调突变（ataxia-telangiectasia mutated, ATM）基因编码的ATM蛋白激酶，随后磷酸化检测点激酶1/2（checkpoint kinase 1/2, CHK1/2）导致下游配体分子p53激活，造成永生化人支气管上皮细胞中S期阻滞^[16]^。在S期阻滞的淋巴瘤细胞中，紫檀芪上调细胞周期CHK2和组蛋白2A变异体（histone family 2A variant, H2AX）磷酸化，下调cyclin A2和CDK2并抑制细胞分裂周期基因25A（cell division cycle gene 25A）表达，抑制肿瘤细胞增殖^[11]^。紫檀芪通过抑制表皮生长因子受体（epidermal growth factor receptor, EGFR）表达，减少其下游信号通路中AKT、mTOR和ERK1/2蛋白磷酸化，导致破坏G_1_期/S期转换，抑制氨基甲酸酯诱导的小鼠肺癌细胞增殖^[15, 28]^。研究表明，紫檀芪下调miR-17、miR-20a和miR-106b表达，恢复第10号染色体缺失的磷酸酶张力蛋白同源物（phosphate and tension homology deleted on chromsome ten, PTEN）表达，进而抑制前列腺癌DU145和22RV1细胞增殖^[15, 29]^。紫檀芪激活腺苷酸活化蛋白激酶（adenosine 5'-monophosphate-activated protein kinase, AMPK）通路并通过减少脂肪酸合酶和乙酰辅酶A羧化酶表达抑制脂肪合成，进而抑制前列腺癌细胞增殖^[1, 30]^。在结肠癌HT-29细胞系中，紫檀芪调控Wnt/β-连环蛋白（β-catenin）信号通路，降低β-catenin、cyclin D1和c-MYC蛋白表达水平，抑制肿瘤细胞增殖^[14]^。此外紫檀芪抗增殖作用机制与下调AKT表达^[2]^和上调锰超氧化物歧化酶（manganese superoxide dismutase, MnSOD）表达有关^[2]^。

## 2 紫檀芪与肿瘤细胞凋亡

凋亡是一种程序性细胞死亡机制，不损伤邻近正常细胞并且降低局部炎症^[31, 32]^，因而激活凋亡是化疗药物抗肿瘤的重要机制^[31, 33]^。紫檀芪具有诱导多种肿瘤细胞发生凋亡的作用，包括膀胱癌^[2, 13]^、肺癌^[2, 7]^、乳腺癌^[1, 2]^、结肠癌^[2, 10]^、前列腺癌^[2, 15]^、胃癌^[2, 27]^、胰腺癌^[34]^、淋巴瘤^[11]^和口腔肿瘤^[12]^等。紫檀芪可以降低线粒体膜电位^[11, 34]^，上调线粒体促凋亡蛋白家族成员Bax^[11, 27]^、Bak^[10]^、Bad^[10, 27]^和Bid^[10, 27]^的表达，下调抗凋亡蛋白B淋巴细胞瘤-2蛋白（B-cell lymphoma-2, Bcl-2）^[11]^和Bcl-xl蛋白^[15]^，进而增加线粒体释放细胞色素C^[34]^和线粒体促凋亡蛋白（second mitochondria-derived activator of caspases, Smac）^[34]^，二者在胞质中激活含半胱氨酸的天冬氨酸蛋白水解酶9（cysteinyl aspartate specific proteinase, caspase 9）和caspase3^[12]^，因而诱导肿瘤细胞发生内源性凋亡。

此外，研究发现紫檀芪处理后可促进T淋巴细胞白血病细胞HUT78^[2, 35]^和胃腺癌细胞（gastric adenocarcinoma cell, AGS）^[2, 27]^自杀相关因子（factor associated suicide, Fas）和Fas配体蛋白表达增加，形成死亡诱导信号复合物（death-inducing signal complex, DISC）激活caspase8，进而活化caspase3、caspase6、caspase7导致细胞调亡^[36]^。此外，紫檀芪可以激活肺癌和食管癌细胞^[8]^发生内质网应激，上调一系列内质网应激相关分子的表达，如磷酸化的RNA依赖的蛋白激酶样内质网激酶（PKR-like ER kinase, PERK）、需肌醇酶1（inositol-requiring kinase 1, IRE1）、转录激活因子4（activating transcription factor 4, ATF4）和CCAAT/增强子结合蛋白同源蛋白（C/EBP homologous protein, CHOP），同时促进钙离子由内质网进入细胞质，进而通过上调Bax和caspase3表达并降低Bcl-2水平导致内质网源性的细胞凋亡^[7]^。研究表明，紫檀芪主要通过抑制蛋白激酶B（protein kinase B, AKT）、哺乳动物雷帕霉素靶蛋白（mammalian target of rapamycin, mTOR）和p70核糖体蛋白S6激酶（p70 ribosomal protein S6 kinase, p70S6K）活性并激活细胞外调节蛋白激酶1/2（extracellular regulated protein kinases 1/2, ERK1/2）诱导膀胱癌T24细胞发生自噬；研究进一步发现，使用自噬抑制剂3-甲基腺嘌呤（3-MA）和巴弗洛霉素A1（Baf A1）预处理后，紫檀芪诱导的肿瘤细胞凋亡增加，细胞活性降低，表明自噬在此过程中抵抗细胞凋亡促进肿瘤细胞存活^[13]^。然而，Ko等^[12]^发现紫檀芪分别与自噬抑制剂3-MA和BafA1共同处理人口腔鳞状细胞癌（squamous cell carcinoma, SAS）和口腔表皮样癌（oral epidermoid carcinoma-Meng 1, OEC-M1）细胞系时，肿瘤细胞的活性大幅增加，提示自噬与凋亡协同作用促进肿瘤细胞死亡。出现以上两种不同结果的原因目前尚不清楚，推测与肿瘤的组织和基因背景不同以及紫檀芪诱导的自噬程度不同有关，低水平的自噬可促进细胞存活，而高水平的自噬则诱导凋亡促进细胞死亡^[4]^。

## 3 紫檀芪与肿瘤侵袭和转移

紫檀芪可抑制三阴性乳腺癌MDA-MB-231和Hs578t细胞系的侵袭，通过上调E-钙粘蛋白（E-cadherin）和抑制锌指转录因子Snail、Slug和ZEB1以及波形蛋白（vimentin）的表达，进而抑制上皮间质转化；此外，紫檀芪显著增加微小RNA（microRNA, miR）-205表达导致Src蛋白表达减少，进而抑制乳腺癌侵袭转移^[18]^。在M2型肿瘤相关巨噬细胞（M2-polarized tumor-associated macrophage, M2 TAM）共培养的乳腺癌MCF7和MDA-MB-231细胞系中，紫檀芪抑制核转录因子-κB（nuclear factor-κB, NF-κB），导致miR-488表达增加，通过上调E-cadherin同时抑制Twist1和vimentin从而抑制乳腺上皮间质转化，降低肿瘤细胞转移特性^[37]^。紫檀芪抑制AKT、ERK1/2和c-Jun氨基末端激酶1/2（c-Jun N-terminal kinase, JNK1/2）磷酸化，进而通过抑制NF-κB、环磷腺苷效应元件结合蛋白（cAMP-response element binding protein, CREB）和SP-1核转运以及基质金属蛋白酶-2（matrix metalloproteinase-2, MMP-2）和尿激酶型纤溶酶原激活剂（urokinase-plasminogen activator, u-PA）的启动子结合活性来抑制MMP-2和u-PA转录表达，抑制口腔鳞状细胞癌SCC-9细胞侵袭^[19]^。研究表明，紫檀芪通过下调蛋白激酶C、表皮生长因子和血管内皮生长因子（vascular endothelial growth factor, VEGF），抑制磷脂酰肌醇-3-羟激酶（phosphoinositide 3 kinase, PI3K）、ERK、p38、JNK和AKT磷酸化，进而抑制NF-κB和激活子蛋白-1（activator protein-1, AP-1）转录因子，导致抑制MMP-9表达，有效抑制对苯二甲酸（terephthalic acid）介导肿瘤转移^[20]^。紫檀芪可促进鼠源性路易斯肺癌（Lewis lung carcinoma）细胞中AKT磷酸化，抑制ERK磷酸化，抑制聚合纤维连接蛋白（poly fibronectin, polyFN）组装，进而抑制癌细胞定植在小鼠肺中^[9]^，提示紫檀芪具有抗肺癌细胞肺转移的能力。研究发现，乳腺癌细胞发生脑转移时，脑转移肿瘤细胞表达高水平c-Met，激活c-Met信号通路可促进肿瘤细胞与脑血管内皮细胞粘附，此外，活化的c-Met可激活丝裂原活化蛋白激酶（mitogen-activated protein kinase, MAPK）诱导白细胞介素1β（interleukin-1β, IL-1β）表达，进而诱导肿瘤相关星形胶质细胞分泌c-Met的配体肝细胞生长因子（hepatocyte-growth factor, HGF），形成了c-Met启动和维持的前馈机制并产生恶性循环，为转移肿瘤细胞创造有利的微环境；而紫檀芪通过降低c-Met mRNA表达来抑制c-Met信号通路，从而抑制肿瘤细胞发生脑转移^[17]^。在乳腺癌MDA-MB-231细胞系中，紫檀芪降低Ras相关C3肉毒素底物1（Ras-related C3 botulinum toxin substrate 1, Rac1）活性，减少威奥综合征蛋白（Wiskott-Aldrich syndrome protein, WASP）家族富含脯氨酸同源蛋白2（WASP/Verprolin homologous protein 2, WAVE2）、肌动蛋白相关蛋白2/3（Actin related protein, Arp2/3）的表达^[38]^，进而抑制肿瘤细胞侵袭和迁移。这些研究证明紫檀芪抑制上皮间质转化、基质金属蛋白酶以及多种信号转导通路具有一定治疗潜力，为控制肿瘤侵袭和转移提供新的治疗方式。

## 4 紫檀芪与肿瘤血管生成

血管生成是促血管生长因子和内源性抗血管生成因子协调作用的复杂过程，例如VEGF和血管抑素（angiostatin）、内皮抑素（endostatin）。正常情况下二者处于平衡状态，而在肿瘤中平衡被打破，激活血管系统，使血管生成过度，例如有血管生成的肿瘤细胞一般比非血管生成肿瘤细胞分泌更多的VEGF^[21]^。研究表明，PI3K/AKT/mTOR通路促进血管生成并在大多数人体肿瘤中激活，活化的PI3K激活AKT，进而磷酸化结节性硬化复合物蛋白2（tuberous sclerosis complex-2, TSC-2），抑制TSC-1/TSC-2复合物生成，从而解除对Ras蛋白脑组织同源类似物（Ras homology enriched in brain, Rheb）的抑制作用，激活mTOR复合物1（mTOR complex 1, mTORC1），进而抑制真核起始因子4E结合蛋白1（eukaryotic translation initiation factor 4E binding protein 1, 4E-BP1），解除对真核起始因子4E（eukaryotic translation initiation factor 4E, eIF-4E）的抑制，促进缺氧诱导因子1α（hypoxia inducible factor 1α, HIF1α）表达，进而诱导VEGF表达，VEGF可与血管内皮细胞作用，增加血管通透性并促进血管内皮细胞增殖，促进血管生成^[21]^，而经紫檀芪处理的膀胱癌细胞中AKT和mTOR活性降低，抑制上述过程，进而抑制肿瘤血管再生^[13]^。此外，紫檀芪处理脑内转移性肿瘤细胞时，通过降低c-Met mRNA表达抑制c-Met，进而抑制MAPK，降低IL-1β表达，从而显著抑制IL-8和趋化因子（c-x-c基序）配体1（chemokine (C-X-C motif) ligand 1, CXCL1）表达，并抑制IL-8和CXCL1介导的白细胞介素8/c-x-c趋化因子受体1（interleukin (IL)-8/C-X-C chemokine receptor 1, CXCR1）信号通路，最终抑制血管生成^[17]^。目前有关紫檀芪在肿瘤血管生成方面作用的研究较少，其作用机制仍需进一步阐明。

## 5 紫檀芪与肿瘤干细胞

研究发现，紫檀芪处理能够减少经γ射线（5 Gy）照射后肝细胞癌CD133（+）Mahlavu细胞中肿瘤干细胞富集，预防肿瘤球形成，减少干性基因表达，增加CD133（+）Mahlavu肿瘤干细胞凋亡并抑制其侵袭和迁移特性^[39]^。在乳腺肿瘤干细胞中，紫檀芪可减少hedgehog蛋白表达从而抑制其与跨膜受体patched相互作用释放smoothened蛋白，进一步抑制PI3K和AKT，因而降低糖原合成酶激酶-3β（glycogen synthase kinase-3β, GSK-3β）蛋白磷酸化，进而增加β-catenin磷酸化并促进其降解，因而导致下游c-Myc和cyclin D1蛋白表达下降并降低细胞表面抗原CD44表达，最终抑制乳腺肿瘤干细胞干性^[22]^。在高侵袭性乳腺癌MDA-MB-231-luc-D3H2LN细胞系中，紫檀芪增加miR-143和miR-200c的表达，导致Ago2表达增加，进而抑制乳腺癌细胞干细胞特性^[15]^；紫檀芪增加多形性成胶质细胞瘤中miR-205表达，导致葡萄糖调节蛋白78（78 kDa glucose regulated protein, GRP78）蛋白表达下降，进而抑制胶质瘤干细胞特性同时增加放疗敏感性^[23]^；紫檀芪通过增加miR-448数量下调NF-κB表达，并抑制上皮间质转化调控蛋白Twist1和vimentin以及增加E-cadherin表达，进而抑制M2型肿瘤相关巨噬细胞诱导的肿瘤干细胞增多和乳腺癌细胞的转移特性^[24]^。值得注意的是，肿瘤微环境中的肿瘤相关巨噬细胞在促进炎症、增殖、免疫编辑和上皮间质转化以及随后的转移中发挥关键作用^[25]^，提示以其为靶向的新的癌症治疗方法。Huang等^[25]^发现，紫檀芪通过下调附膜蛋白粘蛋白1（mucin 1, MUC1）、NF-κB、β-catenin、Sox2和CD133降低M2型肿瘤相关巨噬细胞共培养的肺癌细胞中肿瘤干细胞的百分比，并且抑制肿瘤干细胞的自我更新能力。

## 6 展望

紫檀芪可通过诱导肿瘤细胞凋亡、抑制肿瘤增殖、侵袭和转移、抑制血管生成以及肿瘤干细胞活性等发挥抗肿瘤作用。值得注意的是，在诱导肿瘤细胞凋亡过程中，紫檀芪诱导的自噬可发挥促进和抵抗凋亡两种不同作用^[4]^，然而其分子机制尚未阐明，仍需进一步深入研究。紫檀芪具有显著的抗炎作用，可以抑制皮肤、胰腺和结肠等器官组织的炎症损伤，而炎症在促进肿瘤进展中具有重要作用^[40]^，但紫檀芪的抗炎作用在肿瘤治疗及其机制方面的研究在国内外尚未见报道，值得深入研究。此外，肿瘤干细胞的存在能够启动和维持肿瘤生长^[24]^，而紫檀芪可减少肿瘤干细胞形成并抑制其肿瘤特性和干细胞特性，为肿瘤治疗提供新的开发治疗剂和未来药物的线索。此外，研究^[2]^发现紫檀芪具有抑制细胞色素P450 CYP1A1和CYP1B1催化活性以及清除过氧自由基的作用，从而保护正常细胞对抗致癌物的作用，因此紫檀芪除具有抗肿瘤作用外，还具有预防癌症发生作用。尽管目前大多可获得数据表明紫檀芪具有药理活性且无显著毒性作用，但仍需要更严谨的临床实验，将体外发现与体内生物利用相联系，才能将饮食或治疗剂量的紫檀芪标准化为不同的疗法。因此，紫檀芪的抗肿瘤作用机制及其生物安全性仍需进一步深入研究。
