# 更正声明

**DOI:** 10.3779/j.issn.1009-3419.2020.101.13

**Published:** 2020-03-20

本刊2018年第21卷第12期刊登的题为“紫檀芪抗肿瘤作用机制研究进展”[张晓雁, 张娇, 徐丽群, 等. 紫檀芪抗肿瘤作用机制研究进展. 中国肺癌杂志, 2018, 21(12): 931-936.] 一文中：第931页，英文标题中“Pterostilebene”及英文关键词中“Peterostilbene”应改为“Pterostilbene”。特此更正。对此深表歉意。

Erratum: Emerging Actions of Pterostilbene on Cancer Research

Zhang XY, Zhang J, Xu LQ, *et al*. 

Zhongguo Fei Ai Za Zhi, 2018, 21(12): 931-936. 

In the version of this article initially published, error appeared on page 931. The English title should be “Emerging Actions of Pterostilbene on Cancer Research”. The English key words should be “Pterostilbene; Neoplasms; Apoptosis; Proliferation; Invasion”.

## Notes

https://www.ncbi.nlm.nih.gov/pmc/articles/PMC6318568

